# Loneliness, belonging and psychosomatic complaints across late adolescence and young adulthood: a Swedish cohort study

**DOI:** 10.1186/s12889-024-18059-y

**Published:** 2024-02-29

**Authors:** Karina Grigorian, Viveca Östberg, Jonas Raninen, Sara Brolin Låftman

**Affiliations:** 1 Department of Public Health Sciences, Stockholm University, Stockholm, Sweden; 2 Department of Clinical Neuroscience, Karolinska Institutet, Stockholm, Sweden; 3 Centre for Alcohol Policy Research, La Trobe University, Melbourne, Australia

**Keywords:** Loneliness, Belonging, Psychosomatic complaints, Adolescence, Young adults, Longitudinal, Sweden

## Abstract

**Background:**

Loneliness and belonging refer to social connectedness and are associated with young people’s health; however, the relationship between these constructs and their impact on health is still being discussed. A dual continuum model of belonging and loneliness has been suggested, consisting of four groups depending on the state of loneliness and belonging: socially fulfilled (low loneliness, high belonging), socially indifferent (low loneliness, low belonging), socially searching (high loneliness, high belonging), and socially distressed (high loneliness, low belonging). The aim of this study is to examine loneliness and belonging in a Swedish sample of 17–18-years-olds who were followed over 3 years, and the associations that these aspects share with young people’s psychosomatic complaints during these ages.

**Methods:**

Swedish cohort data collected among late adolescents (age 17–18 in 2019) who were followed up in young adulthood (age 20–21 in 2022) (*n* = 2684) was used to examine the associations between loneliness, belonging, and psychosomatic complaints. Loneliness and belonging were measured by single items and the cross-combinations of these. Three psychosomatic complaints were assessed: stomach ache, headache, and difficulties falling asleep, and a summary index was calculated.

**Results:**

Linear regression analyses showed that loneliness was positively and belonging was negatively cross-sectionally associated with psychosomatic complaints. The socially fulfilled group reported fewer psychosomatic complaints compared to all other groups, while the socially distressed group reported the highest level of psychosomatic complaints. Additional adjustment for sociodemographic characteristics barely affected the estimates. The prospective analysis supported these patterns; however, after adjustment for earlier psychosomatic complaints, the only statistically significant difference in subsequent psychosomatic complaints was found between the socially fulfilled and the socially distressed groups.

**Conclusions:**

Loneliness and belonging (separately and the cross-combinations of these) were cross-sectionally associated with psychosomatic complaints in late adolescence and in young adulthood. Prospectively, only the most vulnerable group in the dual continuum model, the socially distressed group, experienced more psychosomatic complaints than the socially fulfilled group, indicating a temporal relationship. Knowledge about the more nuanced links may be useful for developing specific public health recommendations and interventions for youth, targeting the most vulnerable groups.

**Supplementary Information:**

The online version contains supplementary material available at 10.1186/s12889-024-18059-y.

## Background

Loneliness and belonging both revolve around the concept of social connectedness and represent an individual’s subjective feelings. Loneliness emerges from the mismatch between an individual’s attained and desired social relations and interactions [1]. Being distinct from social isolation, an objective condition characterised by a lack of social contact and social engagement, loneliness represent a subjective feeling of isolation and disconnection from others [2]. It signifies a distressing emotional state characterised by the perception of lacking meaningful social connections and satisfying social interactions. In contrast, belonging refers to the sense of being an integral part of a social group or community, and is linked to experiencing inclusion, acceptance, and a profound sense of connection with others. Belonging represents the fulfilment of the fundamental human need to belong, reflecting an overarching drive to establish and maintain lasting and close interpersonal relationships [3]. It denotes an individual’s successful integration into social networks and meaningful relationships, thus providing a deep sense of fulfilment.

Both loneliness and belonging have been found to be associated with individuals’ health. Loneliness has demonstrated a consistent and strong association with poorer general, physical and mental health [4, 5], including elevated risks of all-cause mortality [6], high blood pressure [7], cardiovascular disease [8], cognitive functioning impairment [9], depression [10], anxiety [10], sleep problems [4], and many other concerns [4]. In a similar way, the inability to fulfil the need for belonging has been linked to long-term health problems and premature mortality [11] in addition to inferior general health, with a particular impact on mental health [12]. In contrast, belonging is associated with fewer health problems and better social and psychological functioning [13, 14].

### The relationships between loneliness and belonging

While typically all individuals share the desire for acceptance and belonging within social groups, the strength of this desire can vary from person to person [15]. There is some evidence to suggest that, depending on the strength of this desire, people may experience loneliness differently (including different impact of loneliness on health) [16]. Indeed, the relation between loneliness and belonging (and their impact on health) is still being discussed: while prior studies suggested that belonging and loneliness can be viewed as opposite ends of a continuum, some recent research has proposed that the fit between perceived loneliness and an individual’s need to belong is critical for an individual’s health [16, 17]. Lim et al. have suggested the dual continuum model of belonging and loneliness where these two constructs are regarded as independent but related, meaning that they can co-exist and occur independently in varying degrees [17]. Four groups of individuals are defined in the model depending on the level of loneliness and belonging, resulting in the socially fulfilled (low loneliness, high belonging), socially indifferent (low loneliness, low belonging), socially searching (high loneliness, high belonging), and socially distressed (high loneliness, low belonging) groups. The socially fulfilled group is expected to demonstrate better health outcomes, while conversely, the socially distressed group is anticipated to encounter the most adverse health conditions.

### Loneliness, belonging, and psychosomatic complaints among young people

Despite the fact that young people generally have a wide range of social connections and are often viewed as being well-integrated into strong social structures, it is not uncommon to feel lonely during this life period. For example, in a study based on data collected in Stockholm, among young people (16–29-year-olds) around three out of ten were bothered by loneliness, and the absolute number of lonely young individuals was greater than in any other age group [18]. Prior research has demonstrated that loneliness tends to increase with age throughout adolescence [19, 20], indicating that older adolescents can be a high-risk group. Furthermore, there is evidence suggesting that young adults tend to experience even higher levels of loneliness than adolescents, making them a particularly vulnerable group [21]. Late adolescence and young adulthood are life stages characterised by many developmental changes and multiple social and relational transitions. Throughout these stages of life, individuals commonly encounter diverse challenges and major social role changes [22] during educational, employment and household transitions. Simultaneously, they navigate the formation of new relationships and attempt to establish self-sufficiency and attain independence in various domains, including education, work, residence, and daily life tasks [22, 23]. These changes can also be accompanied by stress, pressure, and uncertainty and make young individuals exceptionally vulnerable to alterations and problems related to social relationships [24]. The formation of self-determination in young people, being one of the developmental tasks, occurs in a social environment and thrives through positive and supportive social interactions [25]. Undergoing life transitions can additionally result in significant alterations in social relations, affecting the patterns of interactions and social connectedness among individuals. Perceived stress along with factors such as unstable family relations, lack of supportive interpersonal relationships and traumatic relational experiences during these periods can have an impact on young people’s physical and mental health [24, 26]. Furthermore, changes in the social environment may interact with the heightened social sensitivity among young people, including hypersensitivity to the negative consequences of social exclusion [27]. This increased vulnerability can be accompanied by difficulties in establishing new social connections, and, as a result, young people can feel lonelier and experience less belonging [28, 29]. Notably, the manner in which young people cope with changes in their social relations during these life stages can establish prerequisites for their overall adjustment and health later in life.

Additionally, late adolescence and young adulthood are periods of high vulnerability in terms of mental health problems [30], including psychosomatic complaints. These complaints entail health issues lacking an evident medical cause and are indicative of psychosocial stress in young people’s lives [31]. Psychosomatic complaints are common among young people and represent an important public health concern in many countries [32], including Sweden [33]. Nevertheless, while there are numerous studies on loneliness and school belonging, and their association with psychosomatic and other (mental) health complaints during adolescence [14, 19, 34, 35], research on loneliness and belonging in the critical transition from adolescence to young adulthood is scarce.

### Aim of the study

The aim of this study is to examine loneliness and belonging in a Swedish sample of late adolescents (17–18 years) who were followed until young adulthood (20–21 years), and the associations that these aspects of social connectedness share with young people’s psychosomatic complaints during these life stages.

The research questions are:

1. How common are loneliness and belonging at ages 17–18 and 20–21?
2. Are loneliness and belonging cross-sectionally associated with psychosomatic complaints in late adolescence and in young adulthood?
3. Are loneliness and belonging in late adolescence prospectively associated with psychosomatic complaints in young adulthood, even when adjusting for sociodemographic characteristics and earlier psychosomatic complaints?

Gender differences are examined throughout, since previous studies showed that self-reported loneliness and psychosomatic complaints are gendered (in that girls tend to report higher levels of loneliness [19, 20, 34] and psychosomatic complaints than boys [19, 34]), while findings on belonging are mixed [36, 37].

## Methods

### Study design and material

**Fig. 1:**
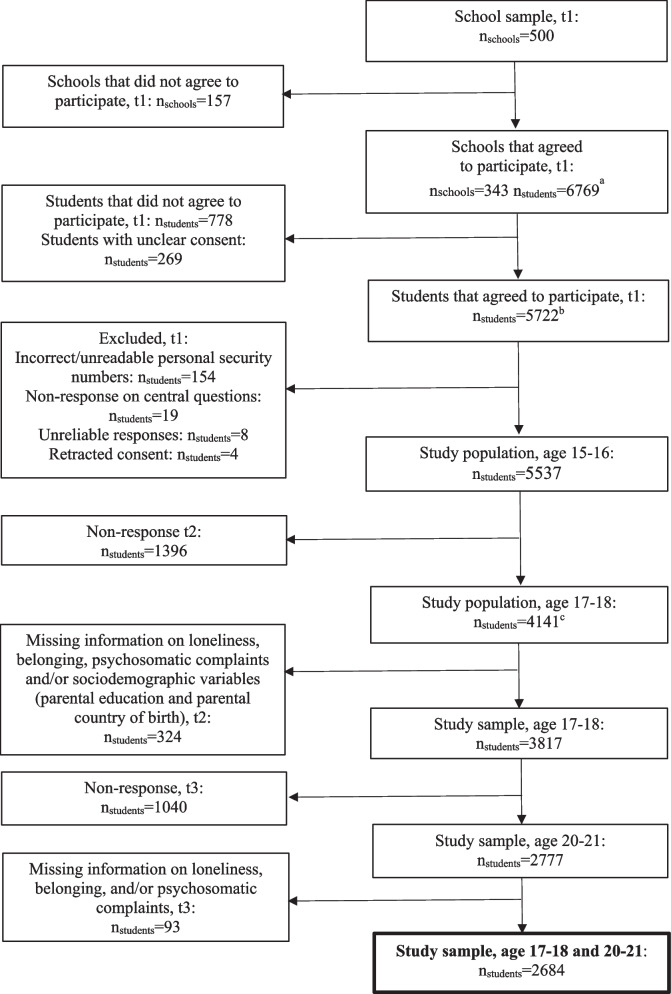
Flow chart of the study sample ^a^ Present at school on the day of the classroom survey ^b^ Responded to the classroom survey ^c^ Responded to the web survey (83%) or the postal survey (17%)

The study uses data from the Futura01 project, which is a Swedish cohort study of a nationwide sample of adolescents attending grade 9 in 2017, most born in 2001 and aged 15 or 16 depending on what month they were born [38]. The first data collection was carried out as a classroom questionnaire during the first half of 2017. For the baseline study, 500 schools across Sweden (and one class in each school) were randomly selected with a response rate of ~ 69% at the school level. There were no statistically significant differences observed between the schools that participated and those that did not participate in terms of grade point average, the proportion of highly educated parents, and the proportion of parents with a foreign background [38]. The second wave was performed in 2019 (when respondents typically attended the second grade of upper secondary school; ~ 17–18 years) as a web or postal survey. The third wave was performed 3 years later, in 2022 (∼20–21 years), as a web survey. The self-report survey data has also been linked to official registers, providing additional information on sociodemographic background characteristics. The measures of loneliness and belonging were introduced at the second and the third waves of data collection. Therefore, only individuals who participated in both the second and the third waves were included in the present study. Among them, only those with complete data on all variables considered in this study were included (*n* = 2684). A flow chart of the sample is displayed in Fig. 1.

### Measures

*Loneliness* was assessed using the question: “Are you bothered by loneliness?” with the response options 1 “Less often than once a month”, 2 “Several or one time a month”, 3 “One time a week”, 4 “Several times a week” and 5 “Each day”. A dichotomous measure was created, where responses from 1 to 3 were coded as 0 (“Low”), while responses from 4 to 5 were coded as 1 (“High”).

*Belonging* was measured by one item on community belonging: “In the past month, how often have you felt like you belonged to a community (e.g., a group of people or an association)?” with the response categories 1 “Never”, 2 “Once or twice a month”, 3 “About once a week”, 4 “About 2–3 times a week”, 5 “Almost every day” and 6 “All days”. The measure was dichotomised with responses from 1 to 3 coded as 0 (“Low”), and responses from 4 to 6 coded as 1 (“High”).

Furthermore, a combination variable was created based on the dichotomous loneliness and belonging measures, representing the interplay between the two dimensions. More specifically, four categories were constructed: socially fulfilled (low loneliness, high belonging), socially indifferent (low loneliness, low belonging), socially searching (high loneliness, high belonging), and socially distressed (high loneliness, low belonging) groups. This categorisation aligns with the dual continuum model of belonging and loneliness represented by Lim et al. [17] and allows to explore how the cross-combinations of loneliness and belonging are linked with psychosomatic complaints.

*Psychosomatic complaints* were assessed by asking participants about the frequency of experiencing stomach aches, headaches and difficulties falling asleep (over the past 6 months) with response options from 1 “Less frequently or never” to 5 “Every day”. Internal consistency was acceptable (Cronbach’s alpha = 0.65 at age 17–18 and Cronbach’s alpha = 0.62 at age 20–21). Summary indices were calculated based on these three items, with a range of 3–15, where higher values represent a greater frequency and co-occurrence of complaints. The same items, which showed strong associations with each other [39], have been used in previous studies to capture psychosomatic complaints [40–44].

Information on* gender* and *birth year* (the vast majority born in 2001) was derived from the participants’ personal identification numbers.

In addition, sociodemographic characteristics that may be associated with both loneliness and belonging and with psychosomatic complaints are adjusted for, i.e., educational/employment status, living arrangements, parental education, and parental country of birth.

*Education/employment status* was based on self-report information. At age 17–18, the categories were “In high school” and “Other”; the latter category including those who marked “Working” (*n* = 17), “Unemployed” (*n* = 20), or did not specify (*n* = 15). At age 20–21, the categories were: “In education”, “Working”, and “Other”; the latter category combining those who had marked “Job seeker” (*n* = 101), “On parental leave” (*n* = 10), “On long-term sick leave” (*n* = 11), “Doing military service” (*n* = 17), “Another activity” (*n* = 63), and others, not specified (*n* = 50).

*Living arrangements* were measured at age 17–18 via the self-report question “How do you live?” with the response alternatives “Lives with both parents”, “Shared residence”, “Lives with one parent”, “Lives in own accommodation” (apart from parents) and “Other”. At age 20–21, the response categories were: “Lives with both parents”, “Lives with one parent”, “Lives in own accommodation (alone)” and “Other” (included those living with siblings, friend(s) or partner and/or partner’s child(ren) and/or own child(ren), and individuals who did not fit into any of the previously mentioned categories).

*Parental education* was based on official register information. The variable indicated the highest educational level attained by the participants’ guardians in 2017 (at the baseline of the Futura01 study) based on four categories: upper secondary school (≤ 2 years) or less, upper secondary school (≥ 3 years), tertiary education (≤ 2 years), and tertiary education (≥ 3 years).

*Parental country of birth* was based on official register information. A variable with two categories was constructed, distinguishing between participants with at least one parent born in Sweden, and those with two parents born outside Sweden.

### Analytical strategy

Descriptive statistics for the sociodemographic variables are provided for the entire sample and stratified by gender. Descriptive statistics were also employed to examine loneliness, belonging and psychosomatic complaints, both for the overall sample and by gender. Gender differences were investigated with χ^2^ tests (for sociodemographic characteristics, loneliness and belonging) and unpaired t-tests (for psychosomatic complaints summary indices). Differences by age (between 17–18 and 20–21 years) were assessed with χ^2^ tests (for loneliness and belonging, and the cross-combinations of these) and with paired t-tests (for psychosomatic complaints). Additionally, transitions between the four groups of loneliness and belonging between ages 17–18 and 20–21 were examined. Subsequently, to investigate the cross-sectional and prospective associations between loneliness and belonging and psychosomatic complaints, ordinary least squares (OLS) regression analysis was employed. In both the cross-sectional and prospective analyses, two approaches were taken: first, loneliness and belonging were analysed separately, and second, the categorisation of loneliness and belonging into the four groups (the socially fulfilled, socially indifferent, socially searching, and socially distressed groups) was examined. Each analysis included one crude model and one or more adjusted models.

In the cross-sectional analyses, depending on the model, adjustments were made for loneliness/belonging (in the analyses where loneliness and belonging were treated separately) and sociodemographic characteristics. In the prospective analyses, we used the lagged dependent variable (LDV) method to control for psychosomatic complaints at age 17–18. The LDV approach, representing a regression analysis controlling for the initial value of the outcome, is widely used for analysing two-wave panel data to offer robust estimates of the influences of independent variables and to test for temporal precedence [45–47]. In the prospective analyses, we also adjusted for loneliness/belonging (in the models where loneliness and belonging were included as distinct variables) and sociodemographic characteristics. To explore potential gender differences in the associations, interaction terms by gender were tested in each model. However, none of these interactions were statistically significant and hence, the results are presented for the full sample, with each model controlling for gender. To account of the clustered nature of the data, where students were nested in classes at the baseline, we estimated robust standard errors, clustering by school class (of which there were a total of 335 classes). To assess the extent of bias resulting from attrition, we additionally compared descriptives of the study sample at age 17–18 with the full sample at age 17–18 and the baseline sample at age 15–16. The statistical analyses were performed using Stata 16.0.

## Results

Sociodemographic characteristics of the study sample are displayed in Table 1. It is seen that 42% of participants were males (*n* = 1116), whereas 58% were females (*n* = 1568). The vast majority of the individuals were in education at age 17–18 (about 98%), while at age 20–21, the number of individuals who studied and who worked were almost equal (about 48% and 43%, respectively). About 65% lived with both parents at age 17–18, whereas 38% did so at age 20–21. Another major change in living arrangements between the two time points was the large increase in ‘Living alone’. About half of the study participants had parents with tertiary education (≥ 3 years) (48.6%) at baseline and a majority of the respondents had at least one parent born in Sweden (85.3%).

**Table 1:** Distribution of sociodemographic characteristics (education/employment status, living arrangements, parental education, and parental country of birth), for all and by gender. Differences by gender assessed with χ^2^ tests. (*n* = 2684)

	All		Males		Females		*P*
	n	%	n	%	n	%	
	2684	100	1116	41.6	1568	58.4	
Educational/employment status (17–18 years)							
In high school	2632	98.1	1084	97.1	1548	98.8	0.015
Other	52	1.9	32	2.9	20	1.2	
Educational/employment status (20–21 years)							
In education	1289	48.0	522	46.8	767	48.9	< 0.001
Working	1143	42.6	459	41.1	684	43.6	
Other	252	9.4	135	12.1	117	7.5	
Living arrangements (17–18 years)							
Both parents	1731	64.5	761	68.2	970	61.9	0.010
Shared residence	301	11.2	120	10.7	181	11.5	
Single parent	397	14.8	138	12.4	259	16.5	
Living in own accommodation	60	2.2	23	2.1	37	2.4	
Other	195	7.3	74	6.6	121	7.7	
Living arrangements (20–21 years)							
Both parents	1031	38.4	506	45.3	525	33.5	< 0.001
Single parent	399	14.9	158	14.2	241	15.4	
Living alone	647	24.1	255	22.9	392	25.0	
Other^a^	607	22.6	197	17.6	410	26.1	
Parental education							
Upper secondary school (≤ 2 years) or less	379	14.1	135	12.1	244	15.6	0.052
Upper secondary school (≥ 3 years)	505	18.8	207	18.5	298	19.0	
Tertiary education (≤ 2 years)	496	18.5	206	18.5	290	18.5	
Tertiary education (≥ 3 years)	1304	48.6	568	50.9	736	46.9	
Parental country of birth							
At least one parent born in Sweden	2288	85.3	958	85.8	1330	84.8	0.462
Both parents born outside Sweden	396	14.7	158	14.2	238	15.2	

^a^Including living with siblings, friend(s) or partner and/or partner’s child(ren) and/or own child(ren)

The results presented in Table 2 showed that 14.5% reported high loneliness at age 17–18, and 17.5% at age 20–21, indicating a statistically significant increase over time (*p* < 0.001). At age 17–18, 78.7% of the participants experienced high belonging, and at age 20–21, the proportion was 75.0%. The decrease over time was statistically significant (*p* < 0.001). At age 17–18, a slightly higher proportion of females than males reported high loneliness (*p* = 0.041). At age 20–21, the gender difference in level of loneliness was not statistically significant (*p* = 0.444). At both time points, females exhibited high belonging less often than males (*p* = 0.001 at age 17–18 and *p* = 0.006 at age 20–21). Examination of the four groups of individuals with different levels of loneliness and belonging in the total sample showed that the socially fulfilled group was the largest (71.7% and 67.4% at ages 17–18 and 20–21 years, respectively), followed by the socially indifferent (13.8% and 15.1%), socially distressed (7.5% and 9.9%), and socially searching groups (7.0% and 7.6%). Over time, the socially fulfilled group exhibited a decline, whereas all other groups experienced a slight increase in size (*p* < 0.001). In terms of gender differences, females were less likely than males to be classified in the socially fulfilled group and more likely to fall into the other three groups. This gender difference was more pronounced at ages 17–18 compared to ages 20–21. Descriptives of the initial (non-dichotomised) scales of loneliness and belonging measures are provided in the Supplementary Material (Table S1). Females reported more psychosomatic complaints compared to males at both ages 17–18 and 20–21. The mean values remained relatively stable over time, both for females and males. Loneliness and belonging by sociodemographic characteristics are displayed in the Supplementary Material (Tables S2 and S3). The sociodemographic characteristics of the full sample at age 15–16 are presented in the Supplementary Material (Table S4). Furthermore, descriptives of loneliness, belonging, psychosomatic complaints and sociodemographic characteristics of the full sample at age 17–18 are provided (Supplementary Material, Table S5). The characteristics of the study sample (in terms of measures of loneliness, belonging, psychosomatic complaints, and sociodemographic characteristics) exhibited no noticeable differences compared with those of the full sample at age 17–18 (see Supplementary Material, Table S5). Although no dramatical differences were observed, comparing Table 1, Tables S4 and S5, we can notice some tendencies in the attrition in that males, those whose parents do not have tertiary education, and those with two parents born outside Sweden were somewhat more likely to drop out across waves.

**Table 2:** Distribution of loneliness, belonging and psychosomatic complaints at ages 17–18 and 20–21. Differences by time assessed with χ^2^ tests (for loneliness and belonging) and with paired t-tests (for psychosomatic complaints) and differences by gender assessed with χ^2^ tests (for loneliness and belonging) and unpaired t-tests (for psychosomatic complaints)

	All (*n* = 2684)				Males (*n* = 1116)				Females (*n* = 1568)				*P*, gender
Loneliness	Low		High		Low		High		Low		High		
17–18 years	85.5		14.5		87.2		12.8		84.4		15.6		0.041
20–21 years	82.5		17.5		83.1		16.9		82.0		18.0		0.444
*P*, time	< 0.001				< 0.001				< 0.001				
Belonging	Low		High		Low		High		Low		High		
17–18 years	21.3		78.7		18.1		81.9		23.6		76.4		0.001
20–21 years	25.0		75.0		22.2		77.8		26.9		73.1		0.006
*P*, time	< 0.001				< 0.001				< 0.001				
Loneliness & Belonging	Socially fulfilled	Socially indifferent	Socially searching	Socially distressed	Socially fulfilled	Socially indifferent	Socially searching	Socially distressed	Socially fulfilled	Socially indifferent	Socially searching	Socially distressed	
17–18 years	71.7	13.8	7.0	7.5	75.5	11.6	6.4	6.5	69.0	15.4	7.4	8.2	0.003
20–21 years	67.4	15.1	7.6	9.9	69.6	13.5	8.2	8.7	65.9	16.1	7.2	10.8	0.048
*P*, time	< 0.001				< 0.001				< 0.001				
Psychosomatic complaints^a^	Mean		SD		Mean		SD		Mean		SD		
17–18 years	7.25		2.73		6.28		2.40		7.94		2.73		< 0.001
20–21 years	7.31		2.65		6.37		2.40		7.97		2.62		< 0.001
*P*, time	0.257				0.231				0.631				

^a^The range of the psychosomatic complaints’ summary index is 3–15

**Fig. 2:**
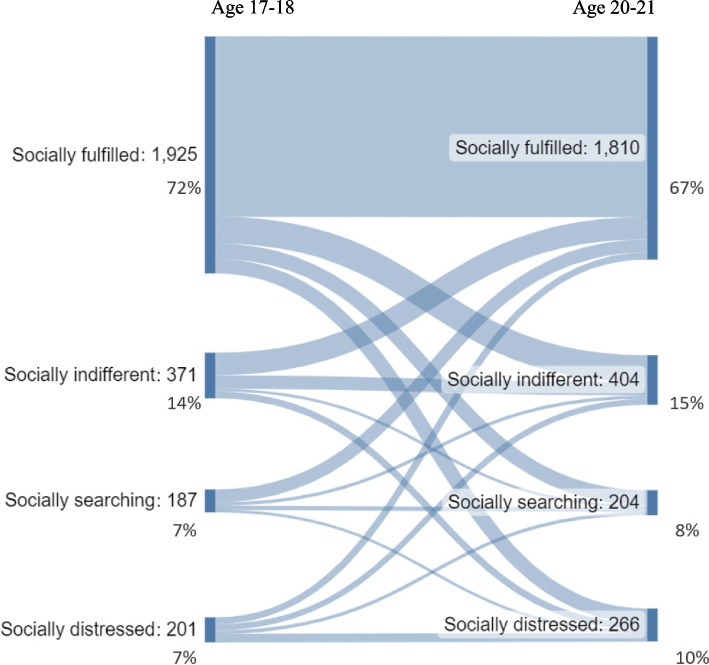
River plot for the transition of four groups categorised as low or high on loneliness and belonging, respectively, from age 17–18 to 20–21. *n* = 2684

Figure 2 presents the transitions between the four groups of loneliness and belonging between ages 17–18 and 20–21. In general, we observed greater stability in the socially fulfilled group in terms of over-time transition, as compared to the other groups. However, we also observed a considerable number of various transitions occurring between the different groups (see Fig. 2).

The findings from the cross-sectional analyses of psychosomatic complaints by loneliness and belonging at ages 17–18 and 20–21 are presented in Table 3. At both time points, loneliness was positively and belonging was negatively associated with psychosomatic complaints. Mutual adjustment for loneliness and belonging resulted in reduced estimates (see Model 1), whilst the estimates showed minimal changes when adding sociodemographic variables (see Model 2).

**Table 3:** Results from cross-sectional analyses of psychosomatic complaints at age 17–18 by loneliness and belonging at age 17–18 (upper panel) and of psychosomatic complaints at age 20–21 by loneliness and belonging at age 20–21 (lower panel). Coefficients from linear regressions and 95% confidence intervals (CI). *n* = 2684

	Psychosomatic complaints (17–18 years)					
	Crude^a^		Model 1^b^		Model 2^c^	
	b	95% CI	b	95% CI	b	95% CI
Loneliness (17–18 years)	**1.58**	1.30; 1.86	**1.33**	1.03; 1.63	**1.25**	0.96; 1.55
Belonging (17–18 years)	**-1.07**	-1.32; -0.82	**-0.72**	-0.98; -0.46	**-0.65**	-0.91; -0.39
	Psychosomatic complaints (20–21 years)					
	Crude^d^		Model 1^e^		Model 2^f^	
	b	95% CI	b	95% CI	b	95% CI
Loneliness (20–21 years)	**1.32**	1.08; 1.57	**0.93**	0.68; 1.19	**0.94**	0.68; 1.19
Belonging (20–21 years)	**-1.29**	-1.54; -1.05	**-1.02**	-1.27; -0.77	**-0.95**	-1.20; -0.70

Bold values denote statistical significance based on the 95% CI

^a^Includes one independent variable at a time (at age 17–18), adjusting for gender

^b^Includes loneliness and belonging (at age 17–18), adjusting for gender

^c^Includes loneliness and belonging (at age 17–18), adjusting for gender, educational/employment status (at age 17–18), living arrangements (at age 17–18), parental education, and parental country of birth

^d^Includes one independent variable at a time (at age 20–21), adjusting for gender

^e^Includes loneliness and belonging (at age 20–21), adjusting for gender

^f^Mutually adjusts for loneliness and belonging (at age 20–21), adjusting for gender, educational/employment status (at age 20–21), living arrangements (at age 20–21), parental education, and parental country of birth

The cross-sectional analyses of psychosomatic complaints and the cross-combinations of loneliness and belonging categorised in the four groups (see Table 4) showed that the socially indifferent, socially searching and socially distressed groups tended to experience more psychosomatic complaints than the socially fulfilled group. The socially distressed group displayed the highest estimates at both age 17–18 and 20–21. In general, the socially indifferent and socially searching groups experienced more psychosomatic complaints than the socially fulfilled group but fewer than the socially distressed group (with the exception of the socially searching group at age 17–18, where the estimates did not significantly differ from those of the socially distressed group). Adjusting for sociodemographic variables had minimal impact on the estimates.

**Table 4:** Results from cross-sectional analyses of psychosomatic complaints at age 17–18 by loneliness and belonging (categorised into four groups) at age 17–18 (upper panel) and of psychosomatic complaints at age 20–21 by loneliness and belonging (categorised into four groups) at age 20–21 (lower panel). Coefficients from linear regressions and 95% confidence intervals (CI). *n* = 2684

Loneliness & belonging (17–18 years)	Psychosomatic complaints (17–18 years)			
	Crude^a^		Model 1^b^	
	b	95% CI	b	95% CI
Socially fulfilled (ref.)	0.00	-	0.00	-
Socially indifferent	***0.86***	0.57; 1.15	***0.78***	0.49; 1.08
Socially searching	**1.59**	1.18; 2.00	**1.49**	1.09; 1.90
Socially distressed	**1.85**	1.45; 2.24	**1.72**	1.33; 2.11
Loneliness & belonging (20–21 years)	Psychosomatic complaints (20–21 years)			
	Crude^c^		Model 1^d^	
	b	95% CI	b	95% CI
Socially fulfilled (ref.)	0.00	-	0.00	-
Socially indifferent	***1.08***	0.80; 1.37	***1.01***	0.72; 1.29
Socially searching	***1.05***	0.70; 1.40	***1.02***	0.66; 1.38
Socially distressed	**1.88**	1.53; 2.23	**1.80**	1.46; 2.13

Bold values denote statistical significance based on the 95% CI

Figures in italics indicate statistically significant difference from the category “Socially distressed” (*p* < 0.05)

^a^Includes the four groups of loneliness and belonging (at age 17–18), adjusting for gender

^b^Includes the four groups of loneliness and belonging (at age 17–18), adjusting for gender, educational/employment status (at age 17–18), living arrangements (at age 17–18), parental education, and parental country of birth

^c^Includes the four groups of loneliness and belonging (at age 20–21), adjusting for gender

^d^Includes the four groups of loneliness and belonging (at age 20–21), adjusting for gender, educational/employment status (at age 20–21), living arrangements (at age 20–21), parental education, and parental country of birth

Table 5 presents the results from the prospective analyses of psychosomatic complaints at age 20–21 by loneliness and belonging at age 17–18. The crude analyses showed that loneliness was positively and belonging was negatively associated with subsequent psychosomatic complaints. In line with the findings of the cross-sectional analyses, including both loneliness and belonging simultaneously (Model 1) reduced the estimates. Further adjustment for socioeconomic variables in Model 2 barely affected the estimates. However, when psychosomatic complaints at age 17–18 were additionally adjusted for, the associations between loneliness and belonging and psychosomatic complaints were substantially attenuated and no longer statistically significant (see Model 3).

**Table 5:** Results from prospective analyses of psychosomatic complaints at age 20–21 by loneliness and belonging at age 17–18. Coefficients from linear regressions and 95% confidence intervals (CI). *n* = 2684

	Psychosomatic complaints (20–21 years)							
	Crude^a^		Model 1^b^		Model 2^c^		Model 3^d^	
	b	95% CI	b	95% CI	b	95% CI	b	95% CI
Loneliness (17–18 years)	**1.07**	0.79; 1.35	**0.88**	0.59; 1.16	**0.84**	0.56; 1.13	0.16	-0.09; 0.41
Belonging (17–18 years)	**-0.77**	-1.01; -0.53	**-0.54**	-0.79; -0.30	**-0.48**	-0.72; -0.23	-0.13	-0.34; 0.09

Bold values denote statistical significance based on the 95% CI

^a^Includes one independent variable at a time, adjusting for gender

^b^Includes loneliness and belonging (at age 17–18), adjusting for gender

^c^Includes loneliness and belonging (at age 17–18), adjusting for gender, educational/employment status (at age 17–18), family structure (at age 17–18), parental education, and parental country of birth

^d^Includes loneliness and belonging (at age 17–18), adjusting for gender, educational/employment status (at age 17–18), living arrangements (at age 17–18), parental education, parental country of birth, and psychosomatic complaints (at age 17–18)

Table 6 presents the results of the prospective analyses of the cross-combinations of loneliness and belonging at age 17–18 and psychosomatic complaints at age 20–21. The crude model showed patterns consistent with those observed in the cross-sectional analyses, and the estimates exhibited minimal alterations after adjustment for sociodemographic variables (see Model 1). However, subsequent adjustment for psychosomatic complaints at age 17–18 revealed that only the association between the socially distressed group at age 17–18 and psychosomatic complaints at age 20–21 remained statistically significant (see Model 2).

**Table 6:** Results from prospective analyses of psychosomatic complaints at age 20–21 by loneliness and belonging (categorised into four groups) at age 17–18. Coefficients from linear regressions and 95% confidence intervals (CI). *n* = 2684

	Psychosomatic complaints (20–21 years)					
	Crude^a^		Model 1^b^		Model 2^c^	
	b	95% CI	b	95% CI	b	95% CI
Loneliness & belonging (17–18 years)						
Socially fulfilled (ref.)	0.00	-	0.00	-	0.00	-
Socially indifferent	***0.52***	0.23; 0.81	***0.45***	0.16; 0.74	0.03	-0.22; 0.28
Socially searching	***0.83***	0.42; 1.23	***0.78***	0.37; 1.18	-0.03	-0.35; 0.30
Socially distressed	**1.45**	1.07; 1.84	**1.35**	0.97; 1.73	**0.42**	0.10; 0.75

Bold values denote statistical significance based on the 95% CI

Figures in italics indicate statistically significant difference from the category “Socially distressed” (*p* < 0.05)

^a^Includes the four groups of loneliness and belonging (at age 17–18), adjusting for gender

^b^Includes the four groups of loneliness and belonging (at age 17–18), adjusting for gender, educational/employment status (at age 17–18), family structure (at age 17-18), parental education and parental country of birth

^c^Includes the four groups of loneliness and belonging (at age 17–18), adjusting for gender, educational/employment status (at age 17–18), living arrangements (at age 17–18), parental education, parental country of birth, and psychosomatic complaints (at age 17–18)

## Discussion

This study explored loneliness and belonging in Swedish youth and their associations with psychosomatic complaints in late adolescence and young adulthood. We analysed loneliness and belonging separately and then examined their cross-combinations as proposed in the dual continuum model of belonging and loneliness [17]. The cross-sectional analyses revealed associations between both loneliness and belonging treated separately and their cross-combinations with current psychosomatic complaints in late adolescence and young adulthood. The prospective analyses further demonstrated an association between the cross-combination of loneliness and belonging (but not loneliness and belonging as distinct constructs) with subsequent psychosomatic complaints.

### Loneliness and belonging among young people in Sweden

While the majority of young people reported relatively low loneliness, approximately 15% of late adolescents and 18% of young adults experienced high loneliness. The prevalence of frequent loneliness among adolescents, as indicated in prior studies conducted in the Nordic countries, was similar or slightly lower [19, 34, 48], with some exceptions [48]. Most of the respondents reported high belonging; nevertheless, approximately 21% of late adolescents and a quarter (25%) of young adults experienced low belonging. Furthermore, loneliness increased from late adolescence to young adulthood, while belonging decreased. These findings are in line with previous studies, highlighting the vulnerability of young people in terms of social connectedness in these life stages [49–51]. It is noteworthy that a majority of the participants were categorised within the socially fulfilled group (low loneliness, high belonging), which exhibited the highest stability in terms of over-time transitions. Nevertheless, there were notable instances of transitions occurring across all four groups, indicating that these groups are subject to considerable variability over time, at least during this period of life.

While our findings indicated that females were slightly lonelier than males in late adolescence, no gender difference was evident in young adulthood. Studies conducted among adolescents in the Nordic countries consistently demonstrated that girls were more likely to experience loneliness than boys [19, 34, 48, 50]. In contrast, a meta-analysis by Maes et al. [52] did not find strong evidence for gender differences in loneliness across the lifespan: even though males tended to be slightly lonelier than females in childhood, adolescence, and particularly in young adulthood, these small gender differences diminished later in life. Therefore, in terms of self-reported loneliness, males and females appeared to be more alike than different [52]. Altogether, the inconsistency of empirical findings regarding gender differences in loneliness has been underscored [52]. In the current study, consistent differences by gender in belonging were found, with females experiencing less belonging than males at both time points. Although the socially fulfilled group prevailed in both genders, it is worth noting that more males than females were categorised within this group, whereas more females than males were categorised within all other groups. Both males and females experienced an increase in loneliness and a decrease in belonging over time.

### The associations between loneliness, belonging and psychosomatic complaints

Regarding our first approach, where we examined loneliness and belonging separately, both of these factors exhibited associations with youth psychosomatic complaints (in most of the models, except for the prospective analysis that adjusted also for earlier psychosomatic complaints). Loneliness was positively and belonging was negatively associated with psychosomatic complaints in both late adolescence (17–18 years) and young adulthood (20–21 years), among males and females alike. Previous studies showed that both loneliness and belonging may affect physical as well as mental health. Loneliness can impact health through multiple mechanisms, including heightened stress levels and the physiological responses associated with stress reactivity [53]. Additionally, loneliness is associated with negative thinking patterns, such as cognitive biases, rumination, and self-criticism, which may result in reduced self-esteem, heightened susceptibility to social threats, and disrupted emotional regulation [54, 55]. Being one of the consequences of loneliness, diminished capacity for self-regulation can ultimately lead to dysfunctional coping including the adoption of unhealthy behavioural patterns and engagement in health-risk behaviours [54]. Belonging can influence health through cognitive and emotional processes, including emotional security and attachment, intimacy, and identification. Furthermore, belonging fosters positive interactions and supportive relationships, which exert both direct and indirect effects on health by enhancing coping abilities and serving as a buffer against the detrimental impact of stress [3]. Hence, loneliness and belonging operate through various mechanisms, some of which may overlap, while others remain unique to each respective construct. It is crucial to note that loneliness can act as both a cause and a consequence of mental health issues, contributing to a self-reinforcing cycle [56]. However, some findings indicate that the effects of loneliness on health tend to manifest earlier, whereas the influence of health on loneliness becomes more prominent later in life [57]. Furthermore, research on social connectedness suggests that the direct association between social connectedness and mental health is more robust and consistently observed than the reversed one [14, 58]. It is also important to mention that in the current study, analyses incorporating both loneliness and belonging in the same model revealed a modest reduction in the respective estimates. This may imply that, while both loneliness and belonging exert independent effects, a part of their association can be attributed to the influence of the other construct. Nevertheless, the estimates were not entirely eliminated, underscoring that loneliness and belonging are interconnected yet distinct constructs. These findings align with the dual continuum model of belonging and loneliness [17], thereby offering support for considering both belonging and loneliness as dual-dimensional concepts.

Consequently, our second approach involved exploring the cross-combinations of loneliness and belonging, leading to the categorisation of the sample into four distinct groups. These results revealed more nuanced associations with psychosomatic complaints. Being socially fulfilled (low loneliness, high belonging) was associated with fewer psychosomatic complaints compared to all other groups, indicating the most favourable condition in relation to such complaints. The combination of low loneliness and high belonging may indicate an optimal situation where social needs are adequately met, and social connectedness is perceived to be strong. Conversely, the socially distressed group (high loneliness, low belonging) exhibited the highest levels of psychosomatic complaints. This combination reflects the least favourable situation with a substantial incongruence between an individual’s social needs and their fulfilment. The socially searching (high loneliness, high belonging) and socially indifferent (low loneliness, low belonging) groups reported higher levels of psychosomatic complaints than the socially fulfilled group but fewer psychosomatic complaints than the socially distressed group. While both the socially fulfilled group and the socially indifferent group are characterised by low loneliness, it appears that low belonging accounts for the higher levels of psychosomatic complaints in the latter group. In addition to the misalignment between social needs and their fulfilment, low belonging may be indicative of a weak motivation to belong, which, in turn, can be associated with psychological dysfunction [59]. This reduced motivation to belong may stem from prior traumatic social experiences, such as repeated rejection or disruption of basic psychological needs for relatedness, competence, and autonomy, leading to a state of learned helplessness [59]. In late adolescence, individuals in the socially searching group reported more psychosomatic complaints compared to those in the socially indifferent group. This finding may be linked to the notion that high loneliness potentially has a more deleterious effect on health compared to low belonging. Finally, our results did not reveal any differences by gender, indicating that the links between the cross-combination of loneliness and belonging and psychosomatic complaints were similar for males and females.

In summary, the dual continuum model revealed that lonely individuals and those experiencing belonging are not homogeneous groups. Furthermore, loneliness does not impact all individuals in the same way, and the alignment between a social situation and an individual’s need to belong can be critical [16]. Moreover, this approach unveils aspects of associations that may remain hidden when only one construct is considered; such as in the prospective analysis of the current study, where no association between loneliness/belonging and psychosomatic complaints was found in the final adjusted model, while the analysis using four distinct groups showed a difference between the socially fulfilled and the socially distressed groups. Also, our findings regarding the cross-combination of loneliness and belonging are similar to those from the study by Beller and Wagner [60], where loneliness and social isolation were examined as two distinct but related indicators of social connectedness. Their findings, along with ours, highlight the importance of various indicators of social connectedness in predicting health, without the superiority of only one construct. The combined effect between high loneliness and low belonging could be explained by common mechanisms leading to poorer health outcomes as well as by mutual influence to each other. Therefore, assessing both constructs is essential for a more comprehensive understanding of the interplay between loneliness and belonging and their association with health outcomes.

### Strengths and limitations

The utilisation of a national sample is one of the strengths of this study as it enhances the robustness of the results and broadens the scope of generalisability. Additionally, the use of longitudinal data enabled the examination of both cross-sectional and prospective associations, offering a more comprehensive understanding of the relationships between loneliness, belonging, and psychosomatic complaints. The time points just before and after high school completion provided an opportunity to study the period of multiple transitions and changes in young people’s lives, which may represent a high-risk phase for increased loneliness, reduced belonging, and heightened psychosomatic complaints. Furthermore, the linkage of the survey data to register data enhanced the reliability of information on sociodemographic covariates used in this study. Finally, the inclusion of measures of both loneliness and belonging allowed for the cross-combination of these aspects and the examination of the dual continuum model of belonging and loneliness in relation to young people’s psychosomatic complaints.

While cohort data offers numerous advantages, attrition remains a concern, introducing potential limitations to the study. Furthermore, survey dropout in longitudinal studies has demonstrated complex associations with diverse health outcomes, the nature of which may vary depending on the specific aspect of health being considered [61]. Therefore, the dropout observed at various stages of the current study might have affected the validity and reliability of the results. Nevertheless, no systematic bias with regards to school-level characteristics was observed between the schools that took part and those that did not participate [38]. There was some systematic bias in the attrition as males, those with parents with less than tertiary education, and those with foreign-born parents were less likely to participate in all waves. Importantly, however, there were no consistent disparities in loneliness, belonging, or psychosomatic complaints at age 17–18 between those who participated in the surveys at both age 17–18 and 20–21, and those who dropped out from the follow-up survey at age 20–21. Nonetheless, it is essential to acknowledge the potential bias resulting from attrition, particularly considering that various health indicators may predict survey dropout [61]. Another limitation pertains to the measurement of loneliness and belonging. In this study, loneliness and belonging were assessed using single items, which is a limitation. Furthermore, direct questions were employed, which asked about perceived loneliness and belonging directly. Additional information based on indirect measures (such as the UCLA (University of California, Los Angeles) 4-item version [62] which is a part of the UCLA loneliness scale [63], The Sense of Belonging Instrument (SOBI) [64], The Challenged Sense of Belonging Scale (CSBS) [65] and others) could be valuable, as it would enable the exploration of different facets of loneliness and belonging and allow for comparisons between direct and indirect assessments [66]. Future studies may consider incorporating both types of measures to provide a more comprehensive examination of loneliness and belonging among young people and their associations with various health outcomes. Additionally, examining various types of belonging may enhance our understanding of the concept during adolescence and young adulthood. Given that young people experience significant social transitions during these years and may change the groups to which they belong and identify with, assessing multiple types of belonging (such as family, school, peer, work, and neighbourhood) and their over-time changes could provide valuable insights. In particular, measuring belonging within specific contexts can provide a more nuanced understanding of this concept among young people. Lastly, while this study measured three common psychosomatic complaints, including a broader list of psychosomatic complaints would allow for the examination of associations with different clusters of youth psychosomatic complaints, such as psychological, somatic, and musculoskeletal (which have demonstrated varying impacts on subsequent mental health [67]). Furthermore, including a broader range of mental health outcomes can enhance our understanding of the similarities and differences between loneliness and belonging in their associations with various mental health issues. For instance, being related but distinct constructs, loneliness and social isolation have demonstrated independent effects on diverse health outcomes [68].

### Policy relevance

In general, a better understanding of the more nuanced links between loneliness and belonging and their association with psychosomatic complaints can inform the development of targeted public health recommendations and interventions for youth. For instance, such knowledge can be valuable for policy development and action plans aiming at strengthening mental health [69] and at preventing and counteracting involuntary loneliness [70, 71]. Previous research has demonstrated that specific interventions can effectively reduce loneliness [72–74]. Social interventions targeting belonging also appear to yield positive effects [59, 75]. Furthermore, belonging commonly has been considered a secondary focus of loneliness reduction interventions rather than a direct target, since supporting belonging has been linked to reduced loneliness [76]. However, prior research has indicated that interventions aimed at reducing loneliness and fostering belonging can differ [72, 77], emphasising the significance of understanding which intervention is more pertinent in specific situations, depending on the target group. Additionally, our study demonstrated that transitions between these four groups were relatively common over these ages. Therefore, given that loneliness and belonging are fluctuant, this life period emerges as an opportune time to implement interventions. Finally, it is also crucial to emphasise initiatives aimed at addressing factors contributing to poor social connectedness. For instance, considering the variations in loneliness and belonging based on education/employment status, as shown in the present study (Tables S2 and S3), a potential strategy to improve young people’s social connectedness could involve reinforcing their opportunities in education and the labour market. Additionally, negative relational experiences, such as abuse and bullying, may lead to impaired social relationships and the avoidance of social relations in the future [78, 79], potentially resulting in loneliness and a diminished belonging. Hence, interventions targeting harmful relations are also pertinent.

## Conclusions

Both loneliness and belonging were associated with psychosomatic complaints among young people. The application of the dual continuum model of belonging and loneliness uncovered more nuanced relationships between these constructs and psychosomatic complaints, some of which remained hidden when analysing each aspect separately. Specifically, the socially fulfilled group exhibited fewer psychosomatic complaints compared to the socially indifferent, socially searching and socially distressed groups, with the latter displaying particular vulnerability regarding young people’s psychosomatic complaints. This underscores the importance of both belonging and loneliness as critical aspects, which, being interrelated, represent distinct facets of an individual’s social connectedness. Interventions targeting loneliness and belonging could be used to reduce mental health issues, specifically psychosomatic complaints, among young people.

### Supplementary Information

**Supplementary Material 1.**

## Data Availability

The data that support the findings of this study are available from Karolinska Institutet but restrictions apply to the availability of these data, which were used under license for the current study, and so are not publicly available. Data are however available from the authors upon reasonable request and with permission of Karolinska Institutet and ethical approval from the Swedish Ethical Review Authority.

## Abbreviations

CI: Confidence Interval
CSBS: Challenged Sense of Belonging Scale
SOBI: Sense of Belonging Instrument
UCLA: University of California, Los Angeles
