# Supplementary material for: Loneliness, belonging and psychosomatic complaints across late adolescence and young adulthood: a Swedish cohort study
Source: BMC Public Health. 2024 Feb 29;24:642. doi: 10.1186/s12889-024-18059-y (PMC10903051; doi:10.1186/s12889-024-18059-y)
Supplement: Supplementary file 1 — Supplementary Material 1. [file 12889_2024_18059_MOESM1_ESM.docx]

**Supplementary Material**

**Table S1** Distribution of loneliness and belonging (original scales) at ages 17-18 and 20-21. Differences by time and differences by gender assessed with χ^2^ tests.

|  | All (n=2684) | | | | | | | | | | Males (n=1116) | | | | | | | | | | Females (n=1568) | | | | | | | | | | p, gender |
| --- | --- | --- | --- | --- | --- | --- | --- | --- | --- | --- | --- | --- | --- | --- | --- | --- | --- | --- | --- | --- | --- | --- | --- | --- | --- | --- | --- | --- | --- | --- | --- |
| Loneliness | Less often than once  a month | | Several or one time  a month | | One time a week | | Several times  a week | | Every day | | Less often than once a month | | Several or one time a month | | One time  a week | | Several times  a week | | Every day | | Less often than once  a month | | Several or one time  a month | | One time a week | | Several times  a week | | Every day | |  |
|  | % | | % | | % | | % | | % | | % | | % | | % | | % | | % | | % | | % | | % | | % | | % | |  |
| 17-18 years | 39.8 | | 25.5 | | 20.3 | | 10.4 | | 4.0 | | 42.4 | | 26.3 | | 18.5 | | 8.9 | | 3.9 | | 37.9 | | 24.9 | | 21.6 | | 11.5 | | 4.1 | | 0.021 |
| 20-21 years | 34.4 | | 27.6 | | 20.5 | | 12.7 | | 4.8 | | 39.5 | | 25.8 | | 17.8 | | 10.5 | | 6.4 | | 30.7 | | 28.8 | | 22.5 | | 14.2 | | 3.8 | | <0.001 |
| p, time | <0.001 | | | | | | | | | | <0.001 | | | | | | | | | | <0.001 | | | | | | | | | |  |
|  |  | | | | | | | | | |  | | | | | | | | | |  | | | | | | | | | |  |
| Belonging | Never | Once or twice a month | | About once  a week | | About 2-3 times  a week | | Almost every  day | | All days | Never | Once or twice | | About once  a week | | About 2-3 times  a week | | Almost every  day | | All days | Never | Once or twice | | About once  a week | | About 2-3 times  a week | | Almost every  day | | All days |  |
| 17-18 years | 7.3 | 6.5 | | 7.5 | | 13.6 | | 28.8 | | 36.3 | 7.4 | 4.7 | | 6.1 | | 13.9 | | 28.9 | | 39.1 | 7.3 | 7.8 | | 8.5 | | 13.5 | | 28.7 | | 34.3 | 0.002 |
| 20-21 years | 8.2 | 8.0 | | 8.8 | | 16.4 | | 28.8 | | 30.0 | 7.8 | 6.5 | | 7.9 | | 16.7 | | 29.2 | | 31.8 | 8.4 | 9.0 | | 9.5 | | 16.1 | | 28.4 | | 28.5 | 0.080 |
| p, time | <0.001 | | | | | | | | | | <0.001 | | | | | | | | | | <0.001 | | | | | | | | | |  |

**Table S2** Loneliness and belonging (dichotomised variables) by sociodemographic characteristics. Differences between groups assessed with χ^2^ tests.

|  | Loneliness | | | | | | | | Belonging | | | | | | | | |  |
| --- | --- | --- | --- | --- | --- | --- | --- | --- | --- | --- | --- | --- | --- | --- | --- | --- | --- | --- |
|  | All (n=2684) | | Males (n=1116) | | | Females (n=1568) | | | All (n=2684) | | | Males (n=1116) | | | Females (n=1568) | | | |
|  | % | p | % | p | | % | p | | % | | p | % | p | | % | p | | |
| 17-18 years |  |  |  | | |  | | |  | | |  | | |  | | | |
| Educational/employment status |  |  |  | |  |  | |  |  | | |  | | |  | | | |
| In high school | 14.1 |  | 12.2 | |  | 15.4 | |  | 79.1 |  | | 82.7 | |  | 76.7 | |  | |
| Other | 32.7 | <0.001 | 34.4 | | <0.001 | 30.0 | | 0.075 | 55.8 | <0.001 | | 56.3 | | <0.001 | 55.0 | | 0.023 | |
| Living arrangements |  |  |  | | |  | | |  | | |  | | |  | | | |
| Both parents | 13.6 |  | 15.8 |  | | 12.2 |  | | 81.1 | |  | 82.5 |  | | 80.1 |  | | |
| Shared residence | 20.9 |  | 20.3 |  | | 21.2 |  | | 71.5 | |  | 76.8 |  | | 68.7 |  | | |
| Single parent | 18.3 |  | 17.4 |  | | 18.9 |  | | 81.7 | |  | 82.6 |  | | 81.1 |  | | |
| Own accommodation | 18.0 | <0.001 | 12.2 | 0.029 | | 21.5 | 0.009 | | 71.3 | | <0.001 | 77.0 | 0.346 | | 67.8 | 0.001 | | |
| Other |  |  |  | | |  | | |  | | |  | | |  | | | |
| Parental education |  |  |  | | |  | | |  | | |  | | |  | | | |
| Upper secondary school (≤2 years) or less | 18.5 |  | 14.1 |  | | 20.9 |  | | 71.8 | |  | 78.5 |  | | 68.0 |  | | |
| Upper secondary school (≥3 years) | 15.6 |  | 13.5 |  | | 17.1 |  | | 76.6 | |  | 80.2 |  | | 74.2 |  | | |
| Tertiary education (≤2 years) | 12.7 |  | 9.7 |  | | 14.8 |  | | 80.2 | |  | 83.0 |  | | 78.3 |  | | |
| Tertiary education (≥3 years) | 13.5 | 0.052 | 13.4 | 0.527 | | 13.6 | 0.044 | | 80.9 | | 0.001 | 82.9 | 0.568 | | 79.4 | 0.002 | | |
| Parental country of birth |  |  |  | | |  | | |  | | |  | | |  | | | |
| At least one parent born in Sweden | 14.3 |  | 12.5 |  | | 15.6 |  | | 80.0 | |  | 83.1 |  | | 77.8 |  | | |
| Both parents born outside Sweden | 15.4 | 0.561 | 14.6 | 0.479 | | 16.0 | 0.875 | | 71.0 | | <0.001 | 74.7 | 0.011 | | 68.5 | 0.002 | | |
|  |  |  |  | | |  | | |  | | |  | | |  | | | |
| 20-21 years |  |  |  | | |  | | |  | | |  | | |  | | | |
| Educational/employment status |  |  |  | | |  | | |  | | |  | | |  | | | |
| In education | 16.7 |  | 17.8 | |  | 16.4 | |  | 77.6 |  | | 79.3 | |  | 76.4 | |  | |
| Working | 15.8 |  | 12.9 | |  | 17.8 | |  | 75.4 |  | | 80.6 | |  | 71.9 | |  | |
| Other | 27.8 | <0.001 | 26.7 | | 0.001 | 29.1 | | 0.004 | 60.3 | <0.001 | | 62.2 | | <0.001 | 58.1 | | <0.001 | |
| Living arrangements |  |  |  | | |  | | |  | | |  | | |  | | | |
| Both parents | 16.0 |  | 14.4 |  | | 17.5 |  | | 74.2 | |  | 78.5 |  | | 70.1 |  | | |
| Single parent | 21.1 |  | 22.2 |  | | 20.3 |  | | 67.4 | |  | 67.7 |  | | 67.2 |  | | |
| Own accommodation (alone) | 22.9 |  | 23.1 |  | | 22.7 |  | | 81.0 | |  | 80.0 |  | | 81.6 |  | | |
| Other^a^ | 12.0 | <0.001 | 10.7 | <0.001 | | 12.7 | 0.002 | | 75.1 | | <0.001 | 81.2 | 0.009 | | 72.2 | <0.001 | | |
| Parental education |  |  |  | | |  | | |  | | |  | | |  | | | |
| Upper secondary school (≤2 years) or less | 20.1 |  | 16.3 |  | | 22.1 |  | | 64.6 | |  | 68.2 |  | | 62.7 |  | | |
| Upper secondary school (≥3 years) | 17.8 |  | 16.9 |  | | 18.5 |  | | 69.7 | |  | 74.4 |  | | 66.4 |  | | |
| Tertiary education (≤2 years) | 16.3 |  | 17.5 |  | | 15.5 |  | | 76.8 | |  | 80.6 |  | | 74.1 |  | | |
| Tertiary education (≥3 years) | 17.1 | 0.501 | 16.7 | 0.993 | | 17.4 | 0.235 | | 79.5 | | <0.001 | 80.3 | 0.009 | | 78.8 | <0.001 | | |
| Parental country of birth |  |  |  | | |  | | |  | | |  | | |  | | | |
| At least one parent born in Sweden | 17.0 |  | 16.2 |  | | 17.5 |  | | 76.8 | |  | 79.1 |  | | 75.2 |  | | |
| Both parents born outside Sweden | 20.7 | 0.070 | 20.9 | 0.143 | | 20.6 | 0.256 | | 64.7 | | <0.001 | 69.6 | 0.008 | | 61.3 | <0.001 | | |

^a^ Including living with siblings, friend(s) or partner and/or partner’s child(ren) and/or own child(ren).

**Table S3** Loneliness and belonging (categorised into four groups) by sociodemographic characteristics. Differences between groups assessed with χ^2^ tests.

|  | All (n=2684) | | | | | Males (n=1116) | | | | | Females (n=1568) | | | | |
| --- | --- | --- | --- | --- | --- | --- | --- | --- | --- | --- | --- | --- | --- | --- | --- |
|  | Socially  fulfilled | Socially indifferent | Socially searching | Socially distressed | p | Socially fulfilled | Socially indifferent | Socially searching | Socially distressed | p | Socially fulfilled | Socially indifferent | Socially searching | Socially distressed | p |
|  | % | % | % | % |  | % | % | % | % |  | % | % | % | % |  |
| 17-18 years |  |  |  |  |  |  |  |  |  |  |  |  |  |  |  |
| Educational/employment status |  |  |  |  |  |  |  |  |  |  |  |  |  |  |  |
| In high school | 72.2 | 13.7 | 7.0 | 7.1 |  | 76.4 | 11.4 | 6.3 | 5.9 |  | 69.3 | 15.3 | 7.4 | 8.0 |  |
| Other | 48.1 | 19.2 | 7.7 | 25.0 | <0.001 | 46.9 | 18.7 | 9.4 | 25.0 | <0.001 | 50.0 | 20.0 | 5.0 | 25.0 | 0.038 |
| Living arrangements |  |  |  |  | <0.001 |  |  |  |  |  |  |  |  |  |  |
| Both parents | 74.4 | 13.0 | 6.2 | 6.4 |  | 77.7 | 11.4 | 5.5 | 5.4 | 0.312 | 71.9 | 14.2 | 6.8 | 7.1 | 0.004 |
| Shared residence | 74.8 | 11.6 | 6.3 | 7.3 |  | 74.2 | 10.0 | 8.3 | 7.5 |  | 75.1 | 12.7 | 5.0 | 7.2 |  |
| Single parent | 61.5 | 17.6 | 10.1 | 10.8 |  | 66.7 | 13.1 | 10.1 | 10.1 |  | 58.7 | 20.1 | 10.0 | 11.2 |  |
| In own accommodation | 70.0 | 11.7 | 11.7 | 6.6 |  | 73.9 | 8.7 | 8.7 | 8.7 |  | 67.6 | 13.5 | 13.5 | 5.4 |  |
| Other | 64.6 | 17.4 | 6.7 | 11.3 |  | 73.0 | 14.9 | 4.0 | 8.1 |  | 59.5 | 19.0 | 8.3 | 13.2 |  |
| Parental education |  |  |  |  |  |  |  |  |  |  |  |  |  |  |  |
| Upper secondary school (≤2 years) or less | 63.6 | 17.9 | 8.2 | 10.3 | 0.015 | 72.6 | 13.3 | 5.9 | 8.2 | 0.892 | 58.6 | 20.5 | 9.4 | 11.5 | 0.021 |
| Upper secondary school (≥3 years) | 69.5 | 14.9 | 7.1 | 8.5 |  | 73.4 | 13.0 | 6.8 | 6.8 |  | 66.8 | 16.1 | 7.4 | 9.7 |  |
| Tertiary education (≤2 years) | 73.6 | 13.7 | 6.7 | 6.0 |  | 78.1 | 12.1 | 4.9 | 4.9 |  | 70.4 | 14.8 | 7.9 | 6.9 |  |
| Tertiary education (≥3 years) | 74.2 | 12.3 | 6.7 | 6.8 |  | 76.1 | 10.5 | 6.9 | 6.5 |  | 72.8 | 13.6 | 6.5 | 7.1 |  |
| Parental country of birth |  |  |  |  |  |  |  |  |  |  |  |  |  |  |  |
| At least one parent born in Sweden | 72.9 | 12.8 | 7.2 | 7.1 | 0.001 | 76.3 | 11.2 | 6.8 | 5.7 | 0.028 | 70.4 | 14.1 | 7.4 | 8.1 | 0.006 |
| Both parents born outside Sweden | 65.2 | 19.4 | 5.8 | 9.6 |  | 70.9 | 14.5 | 3.8 | 10.8 |  | 61.3 | 22.7 | 7.2 | 8.8 |  |
|  |  |  |  |  |  |  |  |  |  |  |  |  |  |  |  |
| 20-21 years |  |  |  |  |  |  |  |  |  |  |  |  |  |  |  |
| Educational/employment status |  |  |  |  |  |  |  |  |  |  |  |  |  |  |  |
| In education | 69.8 | 13.2 | 7.8 | 9.2 |  | 69.9 | 12.3 | 9.4 | 8.4 |  | 69.8 | 13.8 | 6.6 | 9.8 |  |
| Working | 68.8 | 15.4 | 6.6 | 9.2 |  | 74.7 | 12.4 | 5.9 | 7.0 |  | 64.8 | 17.4 | 7.1 | 10.7 |  |
| Other | 49.2 | 23.0 | 11.1 | 16.7 | <0.001 | 51.1 | 22.2 | 11.1 | 15.6 | <0.001 | 47.0 | 23.9 | 11.1 | 18.0 | <0.001 |
| Living arrangements |  |  |  |  |  |  |  |  |  |  |  |  |  |  |  |
| Both parents | 67.4 | 16.6 | 6.8 | 9.2 | <0.001 | 71.2 | 14.4 | 7.3 | 7.1 | <0.001 | 63.8 | 18.7 | 6.3 | 11.2 | <0.001 |
| Single parent | 60.1 | 18.8 | 7.3 | 13.8 |  | 58.2 | 19.6 | 9.5 | 12.7 |  | 61.4 | 18.3 | 5.8 | 14.5 |  |
| In own accommodation (alone) | 68.6 | 8.5 | 12.4 | 10.5 |  | 67.5 | 9.4 | 12.5 | 10.6 |  | 69.4 | 7.9 | 12.2 | 10.5 |  |
| Other^a^ | 71.0 | 17.0 | 4.1 | 7.9 |  | 77.7 | 11.7 | 3.5 | 7.1 |  | 67.8 | 19.5 | 4.4 | 8.3 |  |
| Parental education |  |  |  |  |  |  |  |  |  |  |  |  |  |  |  |
| Upper secondary school (≤2 years) or less | 55.4 | 24.6 | 9.2 | 10.8 | <0.001 | 60.0 | 23.7 | 8.2 | 8.1 | 0.029 | 52.9 | 25.0 | 9.8 | 12.3 | <0.001 |
| Upper secondary school (≥3 years) | 63.8 | 18.4 | 5.9 | 11.9 |  | 67.2 | 15.9 | 7.2 | 9.7 |  | 61.4 | 20.2 | 5.0 | 13.4 |  |
| Tertiary education (≤2 years) | 69.3 | 14.3 | 7.5 | 8.9 |  | 70.9 | 11.6 | 9.7 | 7.8 |  | 68.3 | 16.2 | 5.9 | 9.6 |  |
| Tertiary education (≥3 years) | 71.6 | 11.3 | 7.8 | 9.3 |  | 72.4 | 10.9 | 7.9 | 8.8 |  | 71.1 | 11.5 | 7.7 | 9.7 |  |
| Parental country of birth |  |  |  |  |  |  |  |  |  |  |  |  |  |  |  |
| At least one parent born in Sweden | 68.8 | 14.3 | 8.0 | 8.9 | <0.001 | 70.6 | 13.2 | 8.6 | 7.6 | 0.009 | 67.5 | 15.0 | 7.7 | 9.8 | <0.001 |
| Both parents born outside Sweden | 59.6 | 19.7 | 5.0 | 15.7 |  | 63.9 | 15.2 | 5.7 | 15.2 |  | 56.7 | 22.7 | 4.6 | 16.0 |  |

^a^ Including living with siblings, friend(s) or partner and/or partner’s child(ren) and/or own child(ren).

**Table S4** Descriptives of sociodemographic characteristics (parental education and parental country of birth) in the total sample and stratified by gender. Differences by gender assessed with χ2 tests. Full sample at age 15-16. n=5340 ^a^

|  | All | |  | Males | | Females | | p | |  |
| --- | --- | --- | --- | --- | --- | --- | --- | --- | --- | --- |
|  | n | % |  | n | % | n | % | |  | |
|  | 5340 | 100 |  | 2643 | 49.5 | 2697 | 50.5 | |  | |
| Parental education |  |  |  |  |  |  |  | |  | |
| Upper secondary school (≤2 years) or less | 977 | 18.3 |  | 464 | 17.6 | 513 | 19.0 | |  | |
| Upper secondary school (≥3 years) | 1145 | 21.4 |  | 569 | 21.5 | 576 | 21.4 | |  | |
| Tertiary education (≤2 years) | 948 | 17.8 |  | 460 | 17.4 | 488 | 18.1 | |  | |
| Tertiary education (≥3 years) | 2270 | 42.5 |  | 1150 | 43.5 | 1120 | 41.5 | | 0.365 | |
| Parental country of birth |  |  |  |  |  |  |  | |  | |
| At least one parent born in Sweden | 4398 | 82.4 |  | 2202 | 83.3 | 2196 | 81.4 | |  | |
| Both parents born outside Sweden | 942 | 17.6 |  | 441 | 16.7 | 501 | 18.6 | | 0.070 | |

^a^ Among the 5537 participants, 197 had missing values on parental education and parental country of birth.

**Table S5** Descriptives of loneliness, belonging, psychosomatic complaints (the summary index) and sociodemographic characteristics (educational/employment status, living arrangements, parental education, and parental country of birth) in the total sample and stratified by gender. Differences by gender assessed with χ^2^ tests (for loneliness, belonging and sociodemographic variables) and unpaired t tests (for psychosomatic complaints). Full sample at age 17-18. n=3817

|  | All | |  | Males | | Females | | p | |  |
| --- | --- | --- | --- | --- | --- | --- | --- | --- | --- | --- |
|  | n | % |  | n | % | n | % | |  | |
|  | 3817 |  |  | 1719 | 45.0 | 2098 | 55.0 | |  | |
| Loneliness | 527 | 13.8 |  | 204 | 11.9 | 323 | 15.4 | | 0.002 | |
| Belonging | 3011 | 78.9 |  | 1414 | 82.3 | 1597 | 76.1 | | <0.001 | |
|  |  |  |  |  |  |  |  | |  | |
|  | M | SD |  | M | SD | M | SD | |  | |
| Psychosomatic complaints | 7.24 | 2.73 |  | 6.35 | 2.44 | 7.96 | 2.73 | | <0.001 | |
|  |  |  |  |  |  |  |  | |  | |
|  | n | % |  | n | % | n | % | |  | |
| Educational/employment status |  |  |  |  |  |  |  | |  | |
| In high school | 3711 | 97.2 |  | 1651 | 96.0 | 2060 | 98.2 | |  | |
| Other | 106 | 2.8 |  | 68 | 4.0 | 38 | 1.8 | | <0.001 | |
| Living arrangements |  |  |  |  |  |  |  | |  | |
| Both parents | 2395 | 62.7 |  | 1113 | 64.8 | 1282 | 61.1 | |  | |
| Shared residence | 426 | 11.2 |  | 190 | 11.1 | 236 | 11.3 | |  | |
| Single parent | 607 | 15.9 |  | 248 | 14.4 | 359 | 17.1 | |  | |
| Living in own accommodation | 108 | 2.8 |  | 47 | 2.7 | 61 | 2.9 | |  | |
| Other | 281 | 7.4 |  | 121 | 7.0 | 160 | 7.6 | | 0.144 | |
| Parental education |  |  |  |  |  |  |  | |  | |
| Upper secondary school (≤2 years) or less | 595 | 15.6 |  | 248 | 14.4 | 347 | 16.5 | |  | |
| Upper secondary school (≥3 years) | 778 | 20.4 |  | 351 | 20.4 | 427 | 20.3 | |  | |
| Tertiary education (≤2 years) | 682 | 17.9 |  | 301 | 17.5 | 381 | 18.2 | |  | |
| Tertiary education (≥3 years) | 1762 | 46.1 |  | 819 | 47.7 | 943 | 45.0 | | 0.220 | |
| Parental country of birth |  |  |  |  |  |  |  | |  | |
| At least one parent born in Sweden | 3231 | 84.7 |  | 1470 | 85.5 | 1761 | 83.9 | |  | |
| Both parents born outside Sweden | 586 | 15.3 |  | 249 | 14.5 | 337 | 16.1 | | 0.179 | |
